# Evaluation of Technology-Based Peer Support Intervention Program for Preventing Postnatal Depression: Protocol for a Randomized Controlled Trial

**DOI:** 10.2196/resprot.9416

**Published:** 2018-03-14

**Authors:** Shefaly Shorey, Cornelia Chee, Yap-Seng Chong, Esperanza Debby Ng, Ying Lau, Cindy-Lee Dennis

**Affiliations:** ^1^ National University of Singapore Singapore Singapore; ^2^ National University Hospital Singapore Singapore; ^3^ University of Toronto Toronto, ON Canada

**Keywords:** anxiety, loneliness, peer group, postpartum depression, social support, technology

## Abstract

**Background:**

Multiple international agencies, including the World Health Organization and the International Monetary Fund, have emphasized the importance of maternal mental health for optimal child health and development. Adequate social support is vital for the most vulnerable to postpartum mood disorders. Hence, an urgent need for sustainable social support programs to aid mothers ease into their new parenting role exists.

**Objective:**

This study protocol aims to examine the effectiveness of a technology-based peer support intervention program among mothers at risk for postnatal depression in the early postpartum period.

**Methods:**

A randomized controlled 2-group pretest and repeated posttest experimental design will be used. The study will recruit 118 mothers from the postnatal wards of a tertiary public hospital in Singapore. Eligible mothers will be randomly allocated to receive either the peer support intervention program or routine perinatal care from the hospital. Peer volunteers will be mothers who have experienced self-reported depression and will be receiving face-to-face training to support new mothers at risk of depression. Outcome measures include postnatal depression, anxiety, loneliness, and social support. Data will be collected at immediate postnatal period (day of discharge from the hospital), at fourth week and twelfth week post childbirth.

**Results:**

The recruitment and training of peer support volunteers (N=20) ended in June 2017, whereas recruitment of study participants commenced in July 2017 and is still ongoing. The current recruitment for new mothers stands at 73, with 36 in the control group and 37 in the intervention group. Data collection is projected to be completed by May 2018.

**Conclusions:**

This study will identify a potentially effective and clinically useful method to prevent postnatal depression in new mothers, which is the top cause of maternal morbidity. Receiving social support from others who share similar experiences may enhance the positive parenting experiences of mothers, which in turn can improve the psychosocial well-being of the mothers, tighten mother-child bond, and enhance overall family dynamics for mothers and infants.

**Trial Registration:**

International Standard Randomized Controlled Trial Number ISRCTN14864807; http://www.isrctn.com/ISRCTN14864807 (Archived by WebCite at http://www.webcitation.org/6xtBNvBTX)

## Introduction

The mental well-being of women is highly vulnerable after childbirth, with postpartum mood disorders being the top cause of maternal morbidity [1,2]. The severity of these postnatal affective disorders can range from early maternity blues [3] to postpartum psychosis, which affects less than 1% of mothers [4,5]. Among these affective disorders, postnatal depression (PND) is the most common [5]. Women who have suffered PND often report feelings of inadequacy [6-8], disconnection from others [9,10], role conflicts [8,9,11], loneliness [9], anxiety [11-13], dissatisfaction with life [6,14], insomnia [14], and for severe cases, suicidal ideation [10,15]. Women are susceptible to PND in the first 12 weeks postpartum [16], with the duration dependent on severity [17] and time to onset of the treatment [18]. A total of 50% of clinically depressed mothers have experienced PND symptoms for at least 6 months postpartum [19,20], whereas approximately 25% of untreated mothers experience the symptoms for at least a year [21].

PND poses a huge mental health threat for women, with recent literature reporting an estimated prevalence rate of 10% to 15% for major PND [22,23]. Despite the insufficient statistics in the Singapore context, a recent local study by Shorey et al has discovered that the prevalence of PND symptoms at 4 weeks post birth is at 13% [24]. Additionally, the national G *rowing up in Singapore Towards Health Outcomes* (GUSTO) study [25] has revealed that across the general population, the increased maternal emotional difficulty during pregnancy has adverse effects on child neurodevelopment, in particular, brain regions associated with cognitive and emotional function. These results are supported by other studies that have examined how PND affects the quality of mother-child interactions [26] and has a significant adverse effect on the development of a child [13]. Mothers suffering from PND tend to be more hostile toward the infant [27,28], display disengaged parenting behaviors [27], and are unresponsive toward their infants’ cries [29]. These tendencies increase the risk of attachment anxiety, social behavioral problems [28], and psychopathology [30] in the affected child. Therefore, curbing the risk for depression and anxiety is necessary not only at an individual level but also for the general public health and societal capacity.

### Causes of Postnatal Depression

To date, the causes of PND remain uncertain, with considerable amount of literature suggesting a multifactorial etiology [3,9,31]. However, many studies can identify the primary risk factors for PND. Apart from a history of psychopathology [9,32,33] and demographic variables, such as young age [3], single marital status [32], and low socioeconomic status [3,32], psychosocial variables, such as insufficient social support [14,34], low maternal self-esteem [11,32], marital conflict [14,35], life stress [11,33,34], and childcare stress [9,22,32], are also major predictive factors of PND. The detailed analysis of predictive studies has further revealed underlying issues commonly faced by mothers, which can increase the risk of PND. Common social deficiencies experienced by mothers include insufficient initiated support by others [3,7,22,33], the lack of a close friend to confide in [9], and the inability to find a nonjudgmental person that can listen and empathize with them [36,37].

### Prevention of Postnatal Depression

In previous studies [6,7,14,36,38], emotional and instrumental support have been shown to be important in preventing PND. A previous study has revealed that instrumental support is preferred by mothers more than emotional support [7]. In a study by Gebuza [39], relationship between perceived instrumental support and actual support provided have also been found to be significant, as compared with emotional and informational support. Moreover, the mean social support received from significant others has helped mitigate the decline in mothers’ quality of life after childbirth [39]. Social support through companionship from significant others [6,14,36,38] and family members [3,36,38,40], as well as group belongingness with other identical individuals [36], have also been shown to serve as a protective barrier against PND. Alternatively, in the study of Leahy-Warren et al, no protective relationship was observed between professional support and PND [38]. In addition to social support from family and significant others, the study of Dennis et al also revealed the importance of support from other experienced mothers [36]. New mothers who share experiences with other mothers feel a sense of belonging and reassurance [6,10], which has helped boost their confidence in child rearing and reduced negative feelings [36]. The results from this study suggest that simple intervention means, such as talking to an experienced mother with similar stressors or situation, can act as a buffer against PND [6,10,41].

In addition, numerous randomized controlled trials [42-45] have been conducted to evaluate the effectiveness of psychosocial support interventions to prevent PND. These trial interventions are mostly conducted immediately after post birth [46,47] and primarily targeted high-risk women in professionally led antenatal groups [48,49]. Methodological limitations for these trials include poor measurement of PND and premature timing of outcome assessment. In a large scale Australian trial [47], postoperation debriefing by midwives are not only found ineffective in improving the psychological well-being of mothers but also has actually worsened the emotional health of mothers at 24 weeks. A local trial [50], which evaluated the success of a postpartum psychoeducational program, also found a significant effect in reducing PND. This intervention is administered by midwives paying home visits; therefore, the sustainability of this program is limited by the scarcity of midwives.

### Gaps in the Literature

The scarcity of resources and advancement of technology has led to an increasing trend in telemedicine, which is a previously underutilized platform in the health care sector [51-55]. Technology-based interventions are not only accessible and cost-effective; they also enhance privacy, flexibility, and reduce stigmatization [56,57]. These interventions are especially important for a multiracial, conservative society, such as Singapore, where traditional views and homebound confinement practices restrict new mothers from seeking the necessary help after childbirth [58,59]. Therefore, technology-based support will be the most practical alternative form of support for local mothers. Previous studies have successfully implemented technology-based support intervention for new mothers [51,60]. Technology-based peer support has effectively helped mothers maintain breastfeeding for 3 months postpartum as compared with those in the control group [60]. In another technology-based peer support study [51], pregnant mothers who received support were shown to have decreased anxiety and depressive traits. The risk of PND was also reduced to half of those in the control group. From these studies, we can deduce that the use of technology-based support may be effective in reducing the risk of depression for high-risk mothers.

The aim of this study is to examine the effectiveness of a technology-based peer support intervention program among mothers at risk for postnatal depression in the early postpartum period. This study has 3 objectives:

1. To evaluate the peer support intervention program on maternal outcomes, including PND (primary outcome), anxiety, loneliness, and social support (secondary outcomes)
2. To analyze mothers’ evaluations of their peer support experience
3. To analyze peer volunteers’ evaluations of their peer support experience

We hypothesized that when compared with those in the control group receiving routine care, mothers receiving the peer support intervention program will report a significantly lower level of PND, lower level of anxiety, lower level of loneliness, and higher level of social support received.

## Methods

### Design

A randomized controlled, single-blinded, 2-group pretest and repeated posttest experimental design will be used. Mothers (n=118) recruited from the postnatal wards of a tertiary public hospital will be randomly allocated to 2 groups (intervention group receiving the peer support intervention program and routine perinatal care from the hospital or control group receiving only routine perinatal care from the hospital). Data will be collected at immediate postnatal period (on day of discharge from the hospital), at the fourth week, and the twelfth week post childbirth using Web-based questionnaire surveys that include locally validated and reliable instruments, semistructured face-to-face interviews, and telephone interviews.

### Participants

Potential participants must be mothers who (1) are at least 21 years old, (2) can speak and read English, (3) own a telephone and are willing to share their number, and (4) plan to stay in Singapore for the first 3 months post childbirth. The inclusion criteria for the peer volunteers are mothers who (1) are at least 21 years old, (2) can speak and read English, (3) have delivered healthy baby in the past, (4) have self-reported history of and recovery from PND, (5) have a phone and are willing to share their number and call needy mothers as instructed by the research team, and (6) plan to stay in Singapore for next 6 months from the time of recruitment to participate in the mother-to-mother peer support intervention program. The exclusion criteria for the participants are mothers who (1) have a history or existing psychiatric illnesses, cognitive impairment, “and” or “or” major medical conditions that can interfere with their ability to participate in the study, (2) have had a vacuum- or forceps-assisted delivery with fourth-degree perineal tear; and/or (3) has given birth to a stillborn or a newborn with birth defects and/or medical complications. The exclusion criteria for the peer volunteers are as follows: (1) they have any physical or mental disorders that can interfere with their ability to participate in the study and (2) do not want to share their number and call needy mothers as instructed by the research team.

### Components of the Peer Support Intervention Program

**Figure 1:**
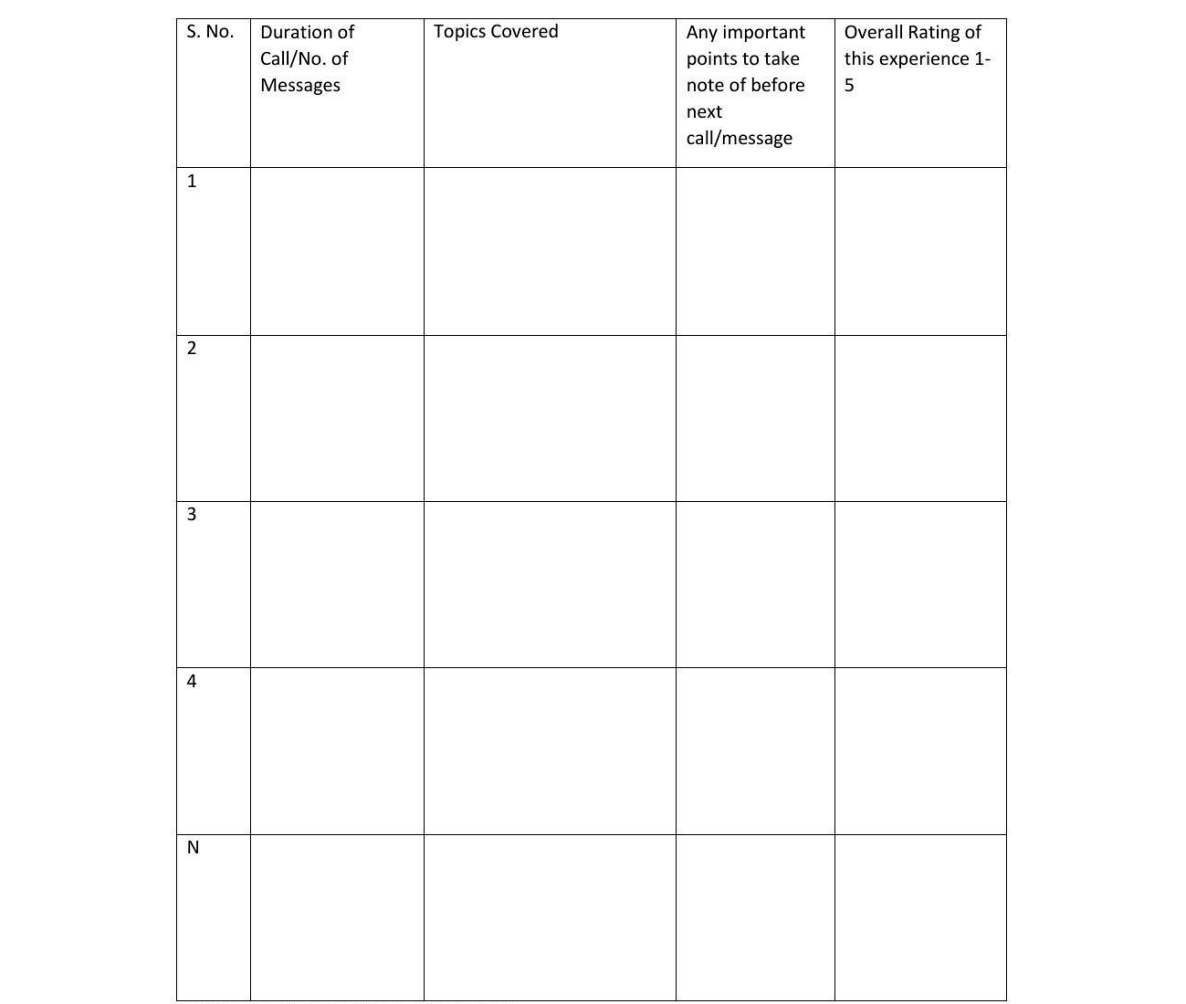
Peer Voluntary Activity Log. Overall rating: 1 least satisfied; 5 most satisfied.

Mothers who participate in the intervention group will receive technology-based peer support alongside routine postnatal care by the hospital, including follow-ups by the obstetrician, nurses, and lactation consultant. Mothers in the control group will only receive standard routine postnatal care provided by the hospital. Research assistant one (RA1) will match the participant to the peer volunteer based on availability [61] and demographics they have provided, especially in the mode of delivery. RA1 will then provide the name and contact details of the participants to the volunteer and inform the participant of the pairing. Each peer volunteer will not only be paired with 1 participant at a given point of time but will also be required to correspond with at least 3 mothers in total. The peer volunteer will initiate contact with the participant within 2 to 3 days post childbirth to discuss suitable timings for future correspondence. On the basis of the effectiveness of previous trials [60,61], peer volunteers will be encouraged to make a minimum of 4 phone contacts in 4 weeks. However, if the dyad cannot compromise on a time for a follow-up call, they will be allowed to correspond through messages or other mobile communication apps such as WhatsApp as frequently as they deem necessary. Frequency and duration will be tailored to maternal needs, which have been found to be effective in the previous trial. As shown in Figure 1, a Peer Volunteer Activity Log will be used to assess the intensity and duration of each intervention session. Additionally, peer support volunteers will be encouraged to maintain a free-text journal on their conversations with the new mothers. Semistructured interviews with peer volunteers and mothers will be conducted to evaluate the nature of the interaction.

### Sample Size Determination

Psychosocial and educational interventions in perinatal period often result in a medium to large effect size on outcome variables [62], such as parenting self-efficacy [24]. Therefore, we can reasonably regard the peer support intervention to have a medium-size effect on the outcome variables. In our study, a 2 sample *t* test will be used to test for differences between the 2 groups. The required sample size to detect a medium effect size of 0.6 (*t* test) at a power of 80% and a significance level of 5% (2-sided) is 45 in each group [63]. In addition, when considering the dropout rate of 30% based on a previous similar study [62] and a preliminary study [24], a minimum sample size of 118 (45×2+45×2×30%=90+28=118) participants, with 59 in each group, is required for this study.

### Randomization

After confirming the eligibility criteria and obtaining the consent of the participants, all mothers will be randomized into 2 groups. Mothers who give consent to participate will be asked to choose a number from 1 to 118 from an opaque envelope. The research randomizer [64] will be used to generate 1 set of 59 numbers. Participants whose picked number matches with any number in the set will be assigned to the intervention group (PIP intervention), and the remaining will be assigned to the control group. The principal investigator, who is not involved in recruitment, intervention, and data collection, will generate the random numbers and will pass them to RA1, who is not involved in data collection.

### Process Evaluation

After the peer support intervention (between 4 and 12 weeks post childbirth), an approximate purposive sample of 20 mothers, 10 each from intervention and control groups (actual number will depend upon data saturation), and all (N=20) peer support volunteers will be selected to participate in the interview to obtain their opinions and comments on the receipt and delivery of the peer support intervention program, respectively. Participation in the interviews will be voluntary. The process evaluation of each interview will take approximately 30-60 min. Participants will be given pseudonyms during the interviews to protect their actual identities. As part of the presentation of the results, their own words will be used in text and will be made anonymous. The interview will be audio-recorded and transcribed into text form. The recruitment will continue until the proposed number is achieved or data saturation is achieved, whichever occurs earlier.

### Outcome Measures and Instruments

The demographic data of mothers (eg, age, gender, ethnicity, education) will be collected. The following instruments will be used to measure the outcomes.

#### Edinburgh Postnatal Depression Scale

The Edinburgh Postnatal Depression Scale (EPDS) [65] is a 10-item scale that has been widely used to measure postnatal depression in international [66,67] and local studies [24,68]. On the basis of previous trials [61,69], the recommended cutoff score of 9 will be used to screen the mothers. A score of >13 is recommended to be used as probable diagnosis for PND. The sensitivity of EPDS ranged from 68% to 80% with specificity of 77% and Cronbach alpha of .88 [65].

#### Patient Health Questionnaire

The Patient Health Questionnaire (PHQ-9) [70] is a shortened 9-item questionnaire extracted from the full PHQ used to diagnose and measure the severity of major depression. It is scored from 0 to 27, with higher scores indicating high severity. An additional item was added to the diagnostic portion for patients who indicated on the questionnaire that they faced problems: “How difficult have these problems made it for you to do your work, take care of things at home, or get along with other people?” The PHQ-9 is well tested and validated in international [71,72] and local [73] studies. The Cronbach alpha in a previous local study was .87 [73].

#### State Trait Anxiety Inventory

The State Trait Anxiety Inventory (STAI) [74] is a 40-item, self-administering 4-point Likert scale used to assess parental anxiety state. This instrument is well tested for its psychometric properties in international [75-77] and local setting [78]. The Cronbach alpha value of the instrument is .8 in local setting [78].

#### University of California, Los Angeles Loneliness Scale

The University of California, Los Angeles (UCLA) Loneliness scale [79] is a 10-item self-report instrument used to measure loneliness. Items are rated on a 4-point Likert-type scale to produce a summative score ranging from 10 to 40, with higher scores indicating higher degrees of loneliness. In a previous study, the Cronbach alpha value was .90 [61].

#### Perceived Social Support for Parenting

This 4-item instrument developed by Leerkes and Crockenberg [80] will be used to measure the satisfaction of parents with the social support from partners and from others. The 5-point scale ranges from 5 to 20 each for the support received from partners or others. The instrument has shown high internal consistency with Cronbach alpha value of .81 in a previous study [80].

A semistructured interview guide will be used for process evaluation. Individual telephone or face-to-face interviews with mothers and peer volunteers will be conducted (4-12 weeks post childbirth) to identify the strengths, weaknesses, and effectiveness of the intervention (from the mothers’ and peer volunteers’ perspectives) as well as on the delivery process from the peer volunteers. All interviews will be audio-recorded.

### Study Procedure

The study will be composed of 2 phases:

Phase 1: Planning intervention strategies for intervention group, including recruitment of peer volunteers, development of peer volunteer training manual, and training the peer volunteers.

Phase 2: Implementing the PIP and investigating its effectiveness on maternal outcomes.

The recruitment of peer volunteers in phase 1 was conducted before the recruitment of participants. Recruitment was done through the blasting of emails to the study venue working community and through word of mouth. On the basis of a previous study [61], 20 peer volunteers were recruited. The recruitment of ethnically diverse peer volunteers was ensured by RA1. RA1 (1) coordinated the peer volunteer recruitment and acquired their informed consent, (2) participated in peer volunteer training sessions, (3) has been pairing mothers with a suitable peer volunteer based on the availability of the volunteer, (4) monitoring the intervention implementation, (5) providing support to peer volunteers as required, and (6) assisting in setting up peer volunteer meetings.

Peer volunteers were required to attend a 4-hour training session conducted by the psychiatrist (one of the study team member). The training for all recruited volunteers was completed in one session in June 2017 to maintain standardization. The training session included roleplaying and discussions to provide necessary skills for the peer-volunteers to successfully deliver the intervention. Peer volunteers were also trained in assessing and conducting the appropriate referrals to relevant health care professionals should the need arise. A peer volunteer training manual was distributed during training to facilitate and guide the intervention process. The trial was introduced during training sessions, and all peer volunteers who agree to participate were required to fill a demographic form and were provided activity logs to account for their sessions with the mothers.

For phase 2, ethics approval has been granted (NHG DSRB: 2017/00815), and participant recruitment has commenced in the postnatal clinics at the study venue. RA1 has informed the nurse managers and clinicians of the respective postnatal wards of the study. The nurse in charge creates a short list of eligible participants based on the selection criteria and verifies the overall physical and psychological well-being of the women. RA1 then approaches those women who meet the inclusion criteria and explains the study purpose and details to them. Voluntary participation is emphasized. Those who are keen to participate are screened using EPDS. On the basis of previous studies [60,61], only mothers with an EPDS score of at least 9 are recruited. After RA1 obtains their written consent, the participants are randomized and grouped into the intervention or control groups accordingly. Participants are then asked to fill a baseline questionnaire, a demographics form, as well as to provide their name and contact information for future data collection and correspondence purposes.

### Data Collection

**Figure 2:**
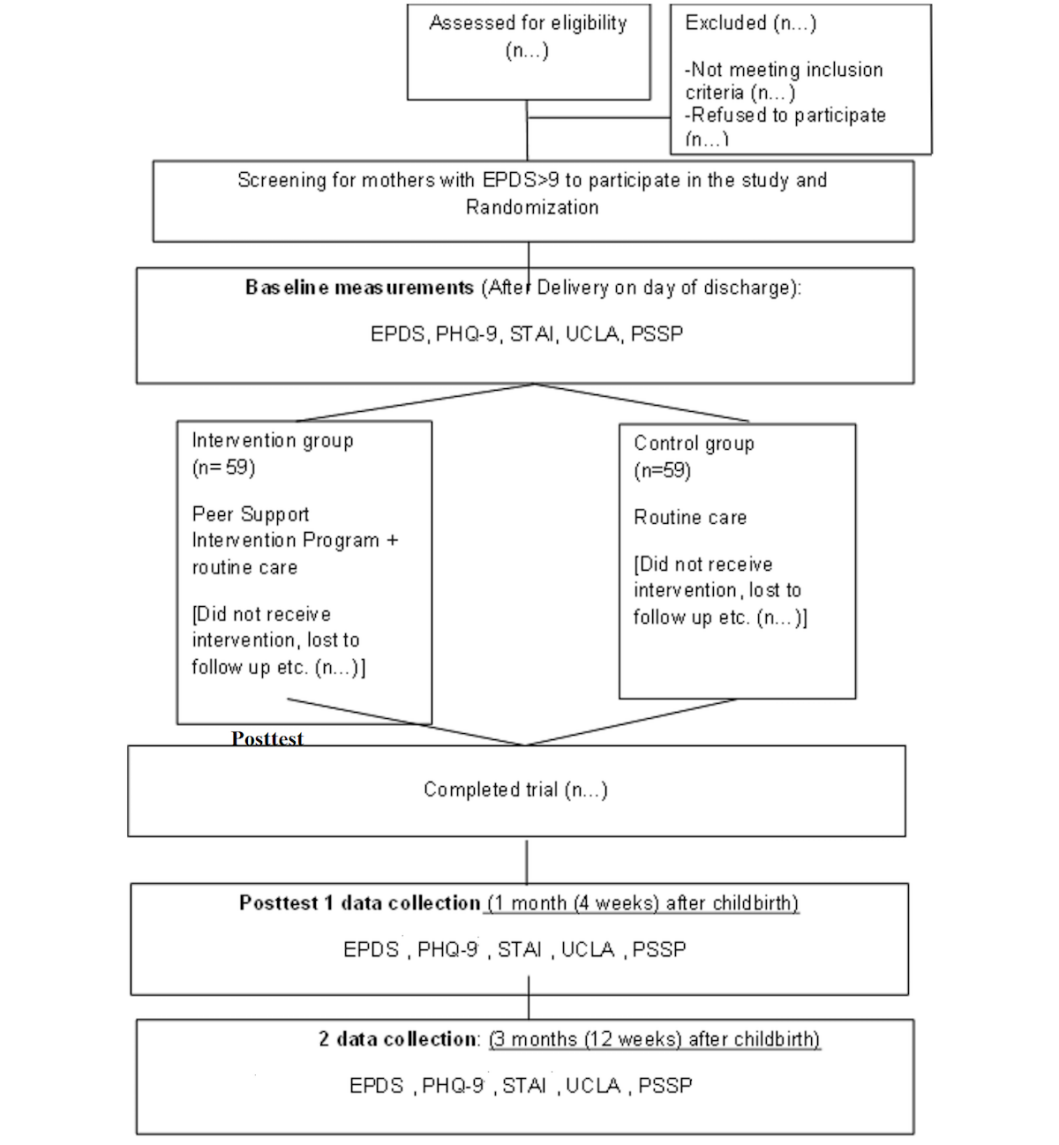
Consolidated Standards of Reporting Trial Flowchart. EPDS: Edinburg Postnatal Depression Scale; PHQ-9: Patient Health Questionnaire 9; STAI: State Trait Anxiety Inventory; UCLA: UCLA Loneliness Scale; PSSP: Perceived Social Support for Parenting.

A single-blinded technique is used. Research assistant two (RA2), who is not part of the randomization and is not involved in the intervention, collects all data and will conduct the process evaluation interviews to avoid bias. Outcome measures of depression, anxiety, loneliness, and perceived social support are collected by RA2 primarily through a Web-based questionnaire, unless the mothers request for an alternative mode, such as through phone call, face-to-face, or via mail. Follow-up time points for all mothers are as follows: (1) immediately after the post birth when discharged from the hospital (baseline), (2) 4 weeks postpartum, and (3) 12 weeks postpartum. Between week 4 and week 12 postpartum, RA2 will invite mothers each from the intervention and control groups to participate in a semistructured process evaluation interview either face-to-face or by telephone. Participation is strictly voluntary. RA2 will also conduct process evaluation interviews with peer volunteers once they are done administering technology-based support to the assigned mothers. The activity logs will also be collected by RA2 for further evaluation. The PND at 12 weeks is chosen as the primary outcome because the results suggest that most PND develop within this time period [19], and we hope to give value to other PND prevention trials [48,49,81-83] by providing comparable results. The 4-week period is chosen for data collection because most of the mothers will be ending their confinement practices, which may alter their support needs [58]. The consolidated standards of the reporting trial flowchart are presented in Figure 2.

### Data Analysis

All quantitative data will be analyzed using the latest version of IBM SPSS. Missing data will be replaced for intention-to-treat analysis. Both intention-to-treat and per-protocol analyses will be conducted to compare the differences between groups. Descriptive statistics, such as mean, standard deviation, and range for continuous data and frequency as well as percentages, will be used for the nominal and ordinal data. Cronbach alpha value will be used to examine the internal consistency of the questionnaires. Inferential statistics, such as independent sample *t* test or analysis of variance (ANOVA), will be used to compare outcome differences among or between the demographic subgroups. Presuming that the outcomes are normally distributed, parametric tests will be used. Repeated measures analysis of covariance (ANCOVA) adjusted for confounding variables (eg, age, education level) will be used to test the effect of intervention on outcomes, including postnatal depression, anxiety, loneliness, and social support across 3 time points in the data collection. The percentage changes in the postnatal depression, anxiety, loneliness, and social support scores from baseline will be calculated for repeated measures ANCOVA. The ANCOVA will be used to test the differences in each outcome among 2 groups at 2 posttests separately.

Qualitative data from the interviews will be analyzed using the thematic analysis [84,85]. The audio interview data will be transcribed verbatim by RA2 concurrently with data collection to capture nonverbal information. Transcribed data will then be sorted into different categories, with similar ideas in a category highlighted in the same color. Related codes or categories will be collated to form subthemes, which will be reviewed and combined to form overarching themes [84] that will describe the opinions of mothers on strengths and weaknesses of the intervention and ways to improve the intervention or on the routine postnatal care. A total of 2 investigators will be involved in the analysis process and will compare and discuss the categories, subthemes, and themes generated and achieved in the consensus. Rigor or trustworthiness, including credibility, transferability, dependability, and conformability, will be considered in the study process [85].

### Ethical Considerations

Ethics approval has been obtained from the National Health Group Domain Specific Review Board before the commencement of the study (Ref number: NHG DSRB: 2017/00185) in April 2017. All mothers who meet the inclusion criteria (EPDS≥9) will be given a set of participant information sheet consisting of a brief introduction, purpose, as well as the advantages and disadvantages of the study conveyed clearly. A written informed consent will be obtained from mothers who agree to participate. The participants are guaranteed anonymity and are informed of their right to withdraw at any point without affecting the subsequent care received. A token of appreciation will be given to all participants.

## Results

Phase 1 of the study has been completed. The peer volunteer manual has been developed, and peer volunteers have been trained. For phase 2, the recruitment of study participants was commenced in July 2017 and is still ongoing. The targeted aim of recruiting 118 mothers will continue for a year. Thus far, a total of 909 participants have been approached and 542 were screened (including those not interested and EPDS score of less than 9). The current sample size to date is 73 mothers (for both the intervention [n=37] and control [n=36] groups). The projected timeline for the completion of data entry and analysis for investigating the effectiveness of the technology-based peer support intervention program to prevent postnatal depression is around May 2019.

## Discussion

Previous local [24,59,86] and international studies [7,10,33,61,87] have shown that mothers have unmet needs during the early postchildbirth period. Given the insufficient support received, many of these mothers are potentially at risk of developing postnatal depression [7,14,24,34]. Results from a systematic review by Dennis [36] on psychosocial intervention methods for postpartum depression have shown that social support is one of the most preferred and effective intervention method to reduce the risk of PND. Previous studies [6,10,36,41] have also shown that additional support from similar other peer volunteers who have also suffered similar symptoms of depression has been proved beneficial for these at-risk, needy mothers. Hence, this study aims to provide technology-based peer support from mothers (peer volunteers) who have suffered and recovered from postnatal depression for mothers at risk of depression with EPDS score more than or equal to 9.

We hope to assess the effectiveness of this peer support intervention program. Optimistically, if the program is effective, it can then be implemented by health care professionals to aid at-risk mothers and reduce the prevalence of postnatal depression. This intervention program may not only be able to introduce a positive parenting experience to new mothers but may also mitigate the adverse psychosocial effects that PND has on the individual, the family, and society in general.

To ensure treatment fidelity, peer volunteers have undergone training to build rapport with their paired mothers, such as through daily text greetings and regular contact through mobile phone or email. Peer volunteers are also trained to contact and reply to the mothers quickly. On the other hand, constant messages and reminders are being sent to the peer volunteers by the research assistant to establish better rapport with them as well.

This study only recruits English-speaking Singaporean mothers, thus limiting its transferability to international settings. However, being set in a multiracial environment can increase international relevance. Additionally, due to practical reasons, the data collection is based on a self-report questionnaire, which is subject to response bias. Recruitment of peer mothers with prior history of postpartum depression also posed as a challenge due to the stigmatization of such mental illnesses in a conservative Asian society. Initial recruitment using posters was ineffective; therefore, recruitment of peer volunteers was mainly done through word of mouth. Despite these challenges and limitations, similar intervention methods in other studies have been shown to be effective.

## Abbreviations

ANCOVA: analysis of covariance
ANOVA: analysis of variance
EPDS: Edinburg Postnatal Depression Scale
GUSTO: Growing Up in Singapore towards Health Outcomes
IBM SPSS: International Business Machines Statistical Package for the Social Sciences
IMF: International Monetary Fund
NHG DSRB: National Health Group Domain Specific Review Board
PHQ-9: Patient Health Questionnaire
PND: postnatal depression
PSSP: perceived social support for parenting
STAI: State Trait Anxiety Inventory
UCLA: University of California, Los Angeles
WHO: World Health Organization

## References

[1] Dennis CL, Stewart DE, Robertson E, Dennis CL, Grace SL, Wallington T (2003). Detection, prevention, and treatment of postpartum depression. Postpartum Depression: Literature Review of Risk Factors and Interventions.

[2] Stocky A, Lynch J (2000). Acute psychiatric disturbance in pregnancy and the puerperium. Baillieres Best Pract Res Clin Obstet Gynaecol.

[3] OHara MW (1995). Postpartum Depression: Causes and Consequences. 1st ed.

[4] VanderKruik R, Barreix M, Chou D, Allen T, Say L, Cohen LS, Maternal Morbidity Working Group (2017). The global prevalence of postpartum psychosis: a systematic review. BMC Psychiatry.

[5] Seyfried LS, Marcus SM (2003). Postpartum mood disorders. Int Rev Psychiatry.

[6] Holopainen D (2002). The experience of seeking help for postnatal depression. Aust J Adv Nurs.

[7] Negron R, Martin A, Almog M, Balbierz A, Howell EA (2013). Social support during the postpartum period: mothers' views on needs, expectations, and mobilization of support. Matern Child Health J.

[8] Choi P, Henshaw C, Baker S, Tree J (2005). Supermum, superwife, supereverything: performing femininity in the transition to motherhood. J Reprod Infant Psychol.

[9] Habel C, Feeley N, Hayton B, Bell L, Zelkowitz P (2015). Causes of women's postpartum depression symptoms: men's and women's perceptions. Midwifery.

[10] McLeish J, Redshaw M (2017). Mothers' accounts of the impact on emotional wellbeing of organised peer support in pregnancy and early parenthood: a qualitative study. BMC Pregnancy Childbirth.

[11] Lazarus K, Roussouw P (2015). Mother's expectations of parenthood: the impact of prenatal expectations on self-esteem, depression, anxiety, and stress post birth. Int J Neuropsychother.

[12] Martell LK (2001). Heading toward the new normal: a contemporary postpartum experience. J Obstet Gynecol Neonatal Nurs.

[13] Beck CT Am J Nurs.

[14] Oates MR, Cox JL, Neema S, Asten P, Glangeaud-Freudenthal N, Figueiredo B, Gorman LL, Hacking S, Hirst E, Kammerer MH, Klier CM, Seneviratne G, Smith M, Sutter-Dallay AL, Valoriani V, Wickberg B, Yoshida K, TCS-PND Group (2004). Postnatal depression across countries and cultures: a qualitative study. Br J Psychiatry Suppl.

[15] Sit D, Rothschild AJ, Wisner KL (2006). A review of postpartum psychosis. J Womens Health (Larchmt).

[16] Cooper PJ, Murray L (1998). Postnatal depression. Br Med J.

[17] Cox JL, Murray D, Chapman G (1993). A controlled study of the onset, duration and prevalence of postnatal depression. Br J Psychiatry.

[18] England S, Ballard C, George S (1994). Chronicity in postnatal depression. Eur J Psychiatry.

[19] Kumar R, Robson KM (1984). A prospective study of emotional disorders in childbearing women. Br J Psychiatry.

[20] Whiffen VE, Gotlib IH (1993). Comparison of postpartum and nonpostpartum depression: clinical presentation, psychiatric history, and psychosocial functioning. J Consult Clin Psychol.

[21] Carpiniello B, Pariante CM, Serri F, Costa G, Carta MG (1997). Validation of the Edinburgh Postnatal Depression Scale in Italy. J Psychosom Obstet Gynaecol.

[22] O'hara MW, Swain AM (2009). Rates and risk of postpartum depression—a meta-analysis. Int Rev Psychiatry.

[23] Brown S, Lumley J (2000). Physical health problems after childbirth and maternal depression at six to seven months postpartum. BJOG.

[24] Shorey S, Chan SW, Chong YS, He HG (2015). A randomized controlled trial of the effectiveness of a postnatal psychoeducation programme on self-efficacy, social support and postnatal depression among primiparas. J Adv Nurs.

[25] Soh S, Chong Y, Kwek K, Saw S, Meaney MJ, Gluckman PD, Holbrook JD, Godfrey KM, GUSTO Study Group (2014). Insights from the Growing Up in Singapore Towards Healthy Outcomes (GUSTO) cohort study. Ann Nutr Metab.

[26] Logsdon MC, Wisner KL, Pinto-Foltz MD (2006). The impact of postpartum depression on mothering. J Obstet Gynecol Neonatal Nurs.

[27] Lovejoy MC, Graczyk PA, O'Hare E, Neuman G (2000). Maternal depression and parenting behavior: a meta-analytic review. Clin Psychol Rev.

[28] Stein A, Pearson RM, Goodman SH, Rapa E, Rahman A, McCallum M, Howard LM, Pariante CM (2014). Effects of perinatal mental disorders on the fetus and child. Lancet.

[29] Murray L, Fiori-Cowley A, Hooper R, Cooper P (1996). The impact of postnatal depression and associated adversity on early mother-infant interactions and later infant outcome. Child Dev.

[30] Oberlander TF, Reebye P, Misri S, Papsdorf M, Kim J, Grunau RE (2007). Externalizing and attentional behaviors in children of depressed mothers treated with a selective serotonin reuptake inhibitor antidepressant during pregnancy. Arch Pediatr Adolesc Med.

[31] Kendall-Tackett K (2010). Depression in New Mothers: Causes, Consequences, and Treatment Alternatives 2nd Edition.

[32] Beck CT (2001). Predictors of postpartum depression: an update. Nurs Res.

[33] Chojenta C, Loxton D, Lucke J (2012). How do previous mental health, social support, and stressful life events contribute to postnatal depression in a representative sample of Australian women?. J Midwifery Womens Health.

[34] Gao LL, Chan SW, Mao Q (2009). Depression, perceived stress, and social support among first-time Chinese mothers and fathers in the postpartum period. Res Nurs Health.

[35] Gotlib IH, Whiffen VE, Wallace PM, Mount JH (1991). Prospective investigation of postpartum depression: factors involved in onset and recovery. J Abnorm Psychol.

[36] Dennis CL, Chung-Lee L (2006). Postpartum depression help-seeking barriers and maternal treatment preferences: a qualitative systematic review. Birth.

[37] Mauthner NS (1997). Postnatal depression: how can midwives help?. Midwifery.

[38] Leahy-Warren P, McCarthy G, Corcoran P (2012). First-time mothers: social support, maternal parental self-efficacy and postnatal depression. J Clin Nurs.

[39] Gebuza G, Kaźmierczak M, Mieczkowska E, Gierszewska M, Banaszkiewicz M (2016). Adequacy of social support and satisfaction with life during childbirth. Pol Ann Med.

[40] Haslam DM, Pakenham KI, Smith A (2006). Social support and postpartum depressive symptomatology: the mediating role of maternal self-efficacy. Infant Ment Health J.

[41] Mead S, Macneil C (2006). Peer support: what makes it unique?. Int J Psychosoc Rehabil.

[42] Armstrong KL, Fraser JA, Dadds MR, Morris J (1999). A randomized, controlled trial of nurse home visiting to vulnerable families with newborns. J Paediatr Child Health.

[43] MacArthur C, Winter HR, Bick DE, Knowles H, Lilford R, Henderson C, Lancashire RJ, Braunholtz DA, Gee H (2002). Effects of redesigned community postnatal care on womens' health 4 months after birth: a cluster randomised controlled trial. Lancet.

[44] Bashour HN, Kharouf MH, Abdulsalam AA, El AK, Tabbaa MA, Cheikha SA (2008). Effect of postnatal home visits on maternal/infant outcomes in Syria: a randomized controlled trial. Public Health Nurs.

[45] Quinlivan JA, Box H, Evans SF (2003). Postnatal home visits in teenage mothers: a randomised controlled trial. Lancet.

[46] Lavender T, Walkinshaw SA (1998). Can midwives reduce postpartum psychological morbidity? A randomized trial. Birth.

[47] Small R, Lumley J, Donohue L, Potter A, Waldenström U (2000). Randomised controlled trial of midwife led debriefing to reduce maternal depression after operative childbirth. Br Med J.

[48] Stamp GE, Williams AS, Crowther CA (1995). Evaluation of antenatal and postnatal support to overcome postnatal depression: a randomized, controlled trial. Birth.

[49] Brugha TS, Wheatley S, Taub NA, Culverwell A, Friedman T, Kirwan P, Jones DR, Shapiro DA (2000). Pragmatic randomized trial of antenatal intervention to prevent post-natal depression by reducing psychosocial risk factors. Psychol Med.

[50] Shorey S, Chan SW, Chong YS, He HG (2015). A randomized controlled trial of the effectiveness of a postnatal psychoeducation programme on self-efficacy, social support and postnatal depression among primiparas. J Adv Nurs.

[51] Bullock LF, Wells JE, Duff GB, Hornblow AR (1995). Telephone support for pregnant women: outcome in late pregnancy. N Z Med J.

[52] Brown R, Pain K, Berwald C, Hirschi P, Delehanty R, Miller H (1999). Distance education and caregiver support groups: comparison of traditional and telephone groups. J Head Trauma Rehabil.

[53] Hellerstedt WL, Jeffery RW (1997). The effects of a telephone-based intervention on weight loss. Am J Health Promot.

[54] Horton R, Peterson MG, Powell S, Engelhard E, Paget SA (1997). Users evaluate LupusLine, a telephone peer counseling service. Arthritis Care Res.

[55] Kaunonen M, Tarkka MT, Laippala P, Paunonen-Ilmonen M (2000). The impact of supportive telephone call intervention on grief after the death of a family member. Cancer Nurs.

[56] Thome M, Alder B (1999). A telephone intervention to reduce fatigue and symptom distress in mothers with difficult infants in the community. J Adv Nurs.

[57] Galinsky MJ, Schopler JH, Abell MD (1997). Connecting group members through telephone and computer groups. Health Soc Work.

[58] Naser E, Mackey S, Arthur D, Klainin-Yobas P, Chen H, Creedy DK (2012). An exploratory study of traditional birthing practices of Chinese, Malay and Indian women in Singapore. Midwifery.

[59] Ong SF, Chan WS, Shorey S, Chong YS, Klainin-Yobas P, He HG (2014). Postnatal experiences and support needs of first-time mothers in Singapore: a descriptive qualitative study. Midwifery.

[60] Dennis CL, Hodnett E, Gallop R, Chalmers B (2002). The effect of peer support on breast-feeding duration among primiparous women: a randomized controlled trial. CMAJ.

[61] Dennis CL, Hodnett E, Kenton L, Weston J, Zupancic J, Stewart DE, Kiss A (2009). Effect of peer support on prevention of postnatal depression among high risk women: multisite randomised controlled trial. BMJ.

[62] Shorey S, Lau Y, Dennis CL, Chan YS, Tam WW, Chan YH (2017). A randomized-controlled trial to examine the effectiveness of the 'Home-but not Alone' mobile-health application educational programme on parental outcomes. J Adv Nurs.

[63] Cohen J (1992). Statistical Power Analysis. Curr Dir Psychol Sci.

[64] (2015). Randomizer.

[65] Cox JL, Holden JM, Sagovsky R (1987). Detection of postnatal depression. Development of the 10-item Edinburgh Postnatal Depression Scale. Br J Psychiatry.

[66] Abiodun OA (1994). A validity study of the Hospital Anxiety and Depression Scale in general hospital units and a community sample in Nigeria. Br J Psychiatry.

[67] Lee AM, Lam SK, Sze Mun Lau SM, Chong CS, Chui HW, Fong DY (2007). Prevalence, course, and risk factors for antenatal anxiety and depression. Obstet Gynecol.

[68] Shorey S, Chan SW, Chong YS, He HG (2015). Predictors of maternal parental self-efficacy among primiparas in the early postnatal period. West J Nurs Res.

[69] Kawachi I, Berkman LF (2001). Social ties and mental health. J Urban Health.

[70] Kroenke K, Spitzer RL, Williams JB (2001). The PHQ-9: validity of a brief depression severity measure. J Gen Intern Med.

[71] Spitzer RL, Kroenke K, Williams JB (1999). Validation and utility of a self-report version of PRIME-MD: the PHQ primary care study. Primary Care Evaluation of Mental Disorders. Patient Health Questionnaire. J Am Med Assoc.

[72] Spitzer RL, Williams JB, Kroenke K, Hornyak R, McMurray J (2000). Validity and utility of the PRIME-MD patient health questionnaire in assessment of 3000 obstetric-gynecologic patients: the PRIME-MD Patient Health Questionnaire Obstetrics-Gynecology Study. Am J Obstet Gynecol.

[73] Sung SC, Low CC, Fung DS, Chan YH (2013). Screening for major and minor depression in a multiethnic sample of Asian primary care patients: a comparison of the nine-item Patient Health Questionnaire (PHQ-9) and the 16-item Quick Inventory of Depressive Symptomatology - Self-Report (QIDS-SR16 ). Asia Pac Psychiatry.

[74] Spielberger CD, Weiner IB, Craighead WE (2010). State-trait anxiety inventory. Corsini Encyclopedia of Psychology.

[75] Andersson L, Sundström-Poromaa I, Wulff M, Aström M, Bixo M (2004). Neonatal outcome following maternal antenatal depression and anxiety: a population-based study. Am J Epidemiol.

[76] Berle JØ, Mykletun A, Daltveit AK, Rasmussen S, Holsten F, Dahl AA (2005). Neonatal outcomes in offspring of women with anxiety and depression during pregnancy. A linkage study from The Nord-Trøndelag Health Study (HUNT) and Medical Birth Registry of Norway. Arch Womens Ment Health.

[77] Fadzil A, Balakrishnan K, Razali R, Sidi H, Malapan T, Japaraj RP, Midin M, Nik JN, Das S, Manaf MR (2013). Risk factors for depression and anxiety among pregnant women in Hospital Tuanku Bainun, Ipoh, Malaysia. Asia Pac Psychiatry.

[78] Cheng TS, Chen H, Lee T, Teoh OH, Shek LP, Lee BW, Chee C, Godfrey KM, Gluckman PD, Kwek K, Saw SM, Chong YS, Meaney M, Broekman BF, Chay OM, Van Bever H, Goh A (2015). An independent association of prenatal depression with wheezing and anxiety with rhinitis in infancy. Pediatr Allergy Immunol.

[79] Russell DW (1996). UCLA Loneliness Scale (Version 3): reliability, validity, and factor structure. J Pers Assess.

[80] Leerkes EM, Crockenberg SC (2002). The development of maternal self-efficacy and its impact on maternal behavior. Infancy.

[81] Chen CH, Wu HY, Tseng YF, Chou FH, Wang SY (1999). Psychosocial aspects of Taiwanese postpartum depression phenomenological approach: a preliminary report. Kaohsiung J Med Sci.

[82] Armstrong KL, Fraser JA, Dadds MR, Morris J (1999). A randomized, controlled trial of nurse home visiting to vulnerable families with newborns. J Paediatr Child Health.

[83] Armstrong KL, Fraser JA, Dadds MR, Morris J (2000). Promoting secure attachment, maternal mood and child health in a vulnerable population: a randomized controlled trial. J Paediatr Child Health.

[84] Braun V, Clarke V (2006). Using thematic analysis in psychology. Qual Res Psychol.

[85] Holloway I, Galvin K (2016). Qualitative Research in Nursing and Healthcare.

[86] Phang KN, Koh SS, Chen HC (2015). Postpartum social support of women in Singapore: A pilot study. Int J Nurs Pract.

[87] Leung S, Arthur DG, Martinson I (2005). Stress in women with postpartum depression: a phenomenological study. J Adv Nurs.

